# Assessing physical literacy with school-aged children in occupational therapy practice: An exploratory qualitative study

**DOI:** 10.1177/03080226231220566

**Published:** 2024-01-05

**Authors:** Emma Clark, Amber Elliott, Alethea Jerebine, Lisa M Barnett

**Affiliations:** 1 School of Health and Social Development, Deakin University, Geelong, VIC, Australia; 2 Centre for Sport, Exercise and Life Sciences, Coventry University, Coventry, UK; 3 Institute for Physical Activity and Nutrition, Deakin University, Geelong, VIC, Australia

**Keywords:** Assessment, clinical practice, paediatric, physical activity

## Abstract

**Introduction:**

Physical literacy (the physical, psychological, social and cognitive capacities needed for movement and physical activity) is arguably important to occupational therapy yet is not explicit within current practice. This study aimed to understand how occupational therapists can engage with the physical literacy concept.

**Method:**

Eight paediatric occupational therapists were recruited by purposive sampling. Participants completed up to three semi-structured interviews to understand perspectives on physical literacy, introduce a novel pictorial assessment and to seek feedback on the assessment after trialling. Interviews were transcribed verbatim and analysed using reflexive thematic analysis.

**Results:**

Sixteen interviews were conducted. Four themes were identified: ‘Foreign tongue’ (occupational therapists have an understanding of physical literacy but used different terminology); ‘Sounds very OT oriented’ (illustrating the connection between physical literacy and occupational therapy); ‘We need the child’s voice’ (measure highlights the child’s perspective); and ‘Contemporary, useful BUT not for all’ (measure had utility for some children but was dependant on child’s condition).

**Conclusion:**

Occupational therapists can apply the physical literacy construct to traditional models and core concepts. The assessment can be used in paediatric occupational therapy practice supported by clinical reasoning, although future tool iterations may need to accommodate some children with disability.

## Introduction

The health benefits of physical activity participation are widely acknowledged, with benefits including increased cardio-respiratory, musculoskeletal, endocrine-metabolic and psychological health and higher quality of life (Brown et al., 2020; Cairney et al., 2019; Caldwell et al., 2020; Fortnum et al., 2018; Pastor-Cisneros et al., 2021). Participation in physical activity also supports general health and wellbeing (Boyt-Schell et al., 2018; Reitz and Scaffa, 2020; World Federation of Occupational Therapists, 2022), as well as aspects of a child’s self-concept (including physical and social self-concept), which are key factors to enjoyment and participation in activity (Goltz and Brown, 2014). Despite this, only 23% of Australian children aged 5–9 years and 15% aged 10–14 years meet the current recommended physical activity guidelines of moderate-vigorous movement for 60 min a day (Australian Government, 2022; World Health Organization, 2020).

**Figure 1.**
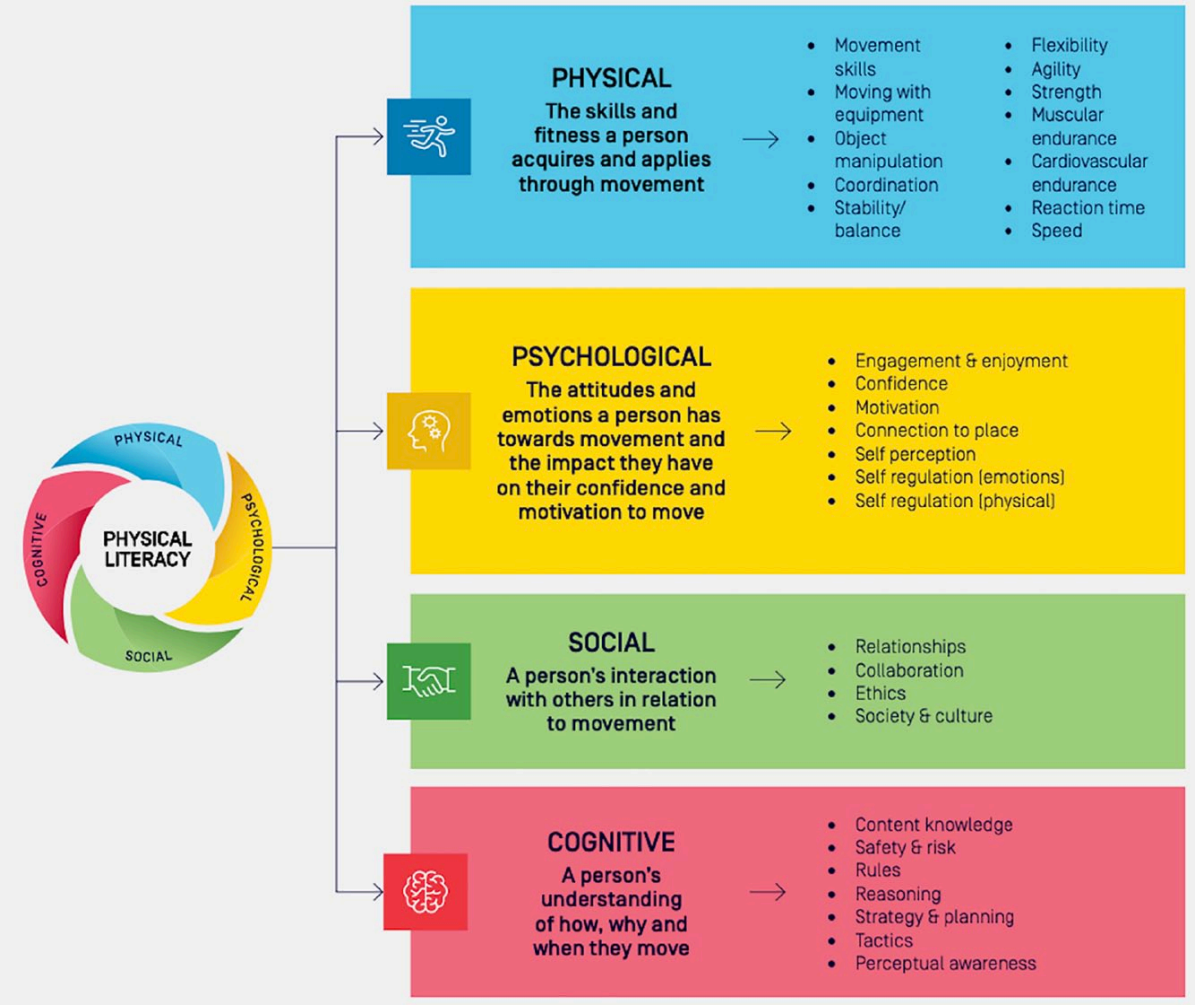
The Australian physical literacy framework: domains (Australian Sports Commission, 2020). Reproduced with permission from ‘Australian Physical Literacy Framework’, by the Australian Sports Commission (2020), https://www.sportaus.gov.au/physical_literacy. Copyright 2020 by the Australian Sports Commission.

Physical literacy is purported to have a formative role in shaping lifelong participation in physical activity. Whitehead (2019, p. 8) conceptualised physical literacy as a holistic construct, describing it as the ‘motivation, confidence, physical competence, knowledge and understanding to value and take responsibility for engaging in physical activities for life’. Whilst this conceptualisation is common, there are many definitions of physical literacy and frameworks of physical literacy in use around the world (Edwards et al., 2017; Martins et al., 2021; Shearer et al., 2018). Some have used consensus making endeavours to reach the resulting definition, such as recent processes in Ireland (Belton et al., 2022) and England (Hurter et al., 2022). The Australian Physical Literacy Framework (APLF) also arose from a consensus process and expanded the original Whitehead definition to also encompass social aspects, that is, ‘the integration of physical, psychological, social and cognitive capabilities, help us live active, healthy and fulfilling lifestyles’ (Australian Sports Commission, 2020). The APLF was the chosen framework for the current research. The APLF covers four domains (physical, psychological, social and cognitive) with 30 elements across these four domains (see Figure 1). For instance, the physical domain refers to the skills and fitness an individual attains and applies via movement, the psychological domain refers to attitudes and emotions an individual has towards movement, the social domain covers an individual’s interaction with others in relation to movement and the cognitive domain includes an individual’s understanding of how, why and when they move (Australian Sports Commission, 2020). The 30 elements are interrelated and considered equally important in the development of physical literacy (Barnett et al., 2022a, 2022b; Australian Sports Commission, 2020).

The construct of physical literacy has been used in research with individuals who have various health and clinical conditions, including intellectual disabilities (Dudley et al., 2016), mental health disorders (Fortnum et al., 2018; Ma et al., 2021) and various medical conditions (Bopp et al., 2019; Do et al., 2021).Whilst the importance of physical literacy is growing in evidence within health, education and sport, there has been relatively little focus of how health professionals engage with the concept and might consider its use in clinical practice for therapy assessment and intervention. Healthcare professionals, including those that work in the allied health sector (e.g. occupational therapists, physiotherapists), play an important role in health promotion. Cornish et al. (2020) reported that health care professionals (e.g. physicians, physiotherapists) are not engaged with the concept of physical literacy due to limited understanding of the concept and a lack of empirical evidence and suggested that further research should investigate how the concept can be used for health promotion, understanding links with health and overall increased participation.

Occupational therapy is a healthcare profession aimed at encouraging children and adults to participate in meaningful occupations by using a holistic approach to promote their overall health and well-being (World Federation of Occupational Therapists, 2022). Occupational therapists working with children will often use a child- and family-centred approach to support participation in childhood occupations and to support development of skills and knowledge (Occupational Therapy Australia, 2016). These childhood occupations often include areas relating to play and social-emotional skills, self-care, learning and education, cognition, motor skills and sensory processing (Novak and Honan, 2019). Since children’s physical literacy development occurs through structured and unstructured physical activity (Cairney et al., 2019; Caldwell et al., 2020), for children aged 5–12 years, it can be considered a part of many childhood occupations involving play and leisure within the community, school or home. Despite physical literacy and occupational therapy both being based upon holistic approaches, there is a gap in the *explicit* use of the construct of physical literacy in occupational therapy practice. To date, we were unable to identify any literature presenting physical literacy understanding within occupational therapy practice. It is possible that occupational therapists do gain information about children’s physical literacy in clinical practice but use other concepts to describe this. Gaining an understanding of how occupational therapists use physical literacy and related concepts in supporting children’s participation in physical activity occupations may be very useful to understand how to best support children’s overall participation.

Within their practice, occupational therapists may gather information using a variety of objective and subjective assessment methods. Using subjective methods allows insight into a child’s personal perspective and provides an opportunity to hear the child’s voice; both of which can often be overlooked when focusing solely upon parent-report (Cordier et al., 2016). Self-report encourages self-regulated learning and self-efficacy and presents the opportunity to empower children to assume ownership of their connection with physical activity and develop their self-awareness (Goss et al., 2022). Current standardised assessments in occupational therapy practice related to physical activity primarily focus on measuring deficiencies in the performance of motor skills (Giblin et al., 2014), which is inherently more of an objective assessment and tends to omit subjective information. Being focused on the physical domain, these assessments are unable to provide a comprehensive and holistic assessment of all elements of physical literacy, and child focused subjective assessments may be required to provide a complete physical literacy profile on an individual. Incorporating self-report, subjective assessments into paediatric occupational therapy practice speaks to child-centred practice, which is a crucial element of working with children. With many assessments measuring objective skills, or seeking caregiver insight, opportunity to hear from the child themselves is lacking. To support our fundamental beliefs of person-first practice, exploring and considering diverse tools to gather this information is pertinent to all paediatric occupational therapists.

This study sought to explore the (i) current knowledge of physical literacy within occupational therapy practice, (ii) relationship between physical literacy and occupational therapy, (iii) feasibility of the use of a pictorial self-report physical literacy tool designed to match the APLF in paediatric occupational therapy practice and (iv) the value of physical literacy self-assessment in occupational therapy practice. The findings can contribute to the evidence base within occupational therapy, which may, in turn, inform programmes and policies to improve physical literacy in Australian children and promote health and well-being from a young age through participation in physical activity.

## Methodology

### Study design

This study adopted an interpretive phenomenological approach to explore and interpret the perspectives of paediatric occupational therapists. Interpretive phenomenological analysis (IPA) is a methodological approach used in health research to investigate the subjective experiences of individuals, including healthcare professionals (Peat et al., 2019).Through in-depth reflective inquiry, IPA seeks to explore and understand what a lived experience means to an individual (Peat et al., 2019). Ethics approval was obtained from Deakin University Human Ethics Advisory Group – Health (Approval No. HEAG-46-2022).

### Participants and recruitment

Paediatric occupational therapists were recruited through purposive sampling. Inclusion criteria included occupational therapists who were (i) practising in Victoria, Australia; (ii) practising with school-aged children (aged 5–12 years) within the last 5 years; (iii) registered with the Australian Health Practitioner Regulation Australia and (iv) provided informed consent to participate. Participants were recruited through occupational therapy providers within the industry and relevant online interest groups in Victoria. Plain language statements were provided to participants to clearly outline the purpose and expectations of the research study, including participation requirements and the consent/withdrawal of consent process.

### Assessment instrument used in testing

**Figure 2.**
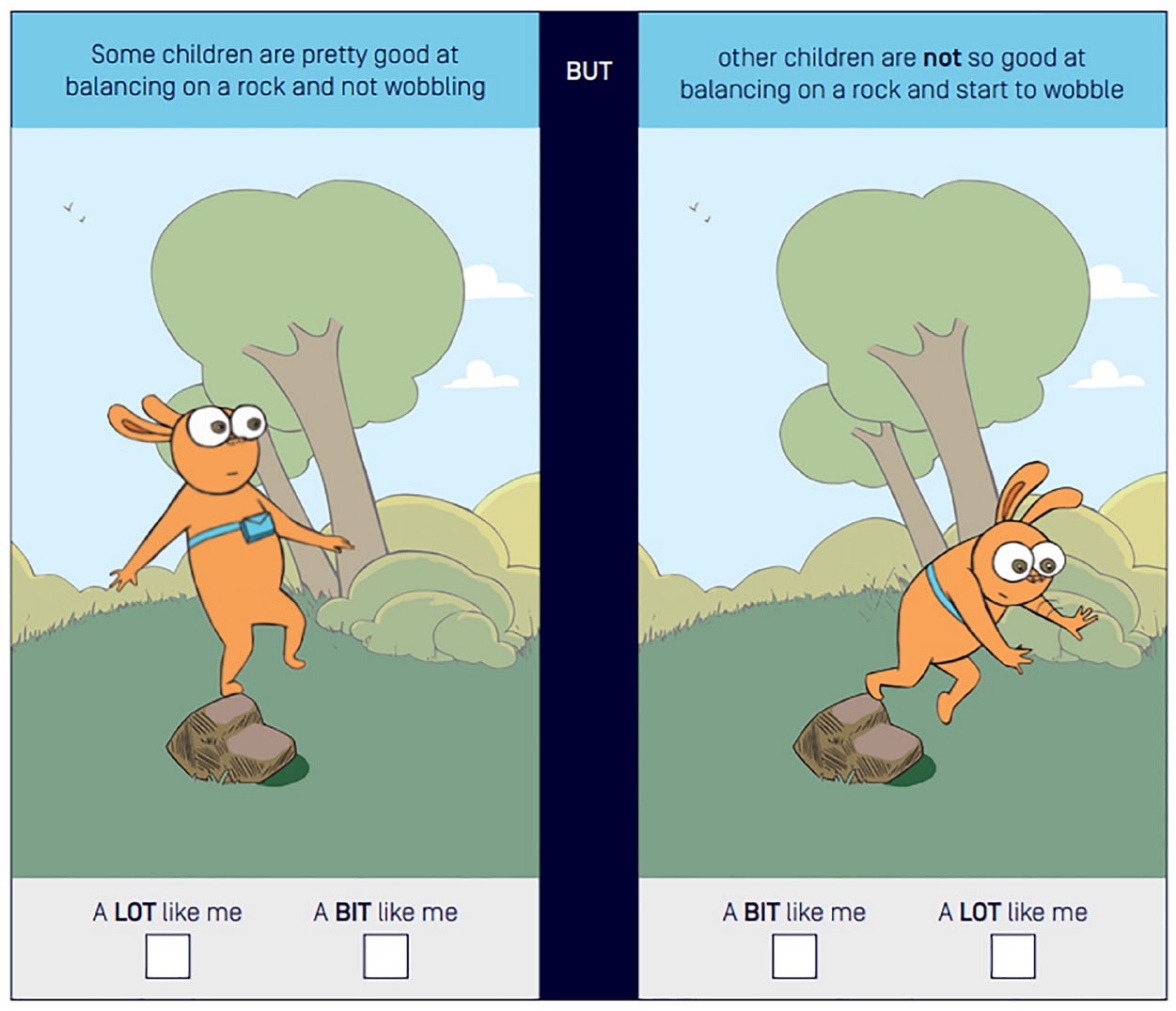
Example item of Physical Literacy in Children Questionnaire survey (Sport Australia, 2021). Reproduced with permission from ‘Physical literacy in children questionnaire: user guide’, by Sport Australia (2021). https://www.sportaus.gov.au/__data/assets/pdf_file/0009/994248/Physical-Literacy-in-Children-Questionnaire-User-Guide.pdf. Copyright 2021, Sport Australia.

The Physical Literacy in Children Questionnaire (PL-C Quest) is a self-report pictorial assessment designed for use by school-aged children (5–12 years) (Sport Australia, 2021). The PL-C Quest was chosen for this study as it was designed to map to the Australian Framework of Physical Literacy and promotes a holistic child-centred approach (Barnett et al., 2022a, 2022b). It includes 30 questions (matched to the 30 items of the APLF) and takes approximately 12 min to complete for typically developing children aged 7–12 years (Barnett et al., 2022a, 2022b). The child examines two images, one image with a rabbit character performing the physical literacy task well and one image with the character performing it not so well. The child has to pick which image is like them and then they are asked whether the image is a lot like them, or a bit like them, resulting in a four-point response scale. For example, see Figure 2. There are two versions of the PL-C Quest developed for use with: (i) younger children, typically developing 4–8-year old’s or those who require assistance with reading and (ii) older children, typically developing 8–12-year olds (Barnett et al., 2022a, 2022b; Sport Australia, 2021). There are words to accompany each of the images that are either read out to the younger children or self-read for older children. The PL-C Quest has evidence for construct validity and internal consistency in typically developing children aged 7–12 years (Barnett et al., 2022a, 2022b) and was identified as one of the physical literacy instruments with more evidence of validity data in a recent review (Barnett et al., 2023). However, it has not yet been used or tested within a clinical context or with non-typically developing children.

### Data collection

Eight occupational therapists participated in semi-structured online interviews; first to understand their perspectives on physical literacy, and subsequently (as required) to discuss their experience trialling the PL-C Quest with children in their practice. The semi-structured interview was piloted with two occupational therapists, both with a minimum of 13 years clinical experience, on two separate occasions to refine overall interview design and process, as well as piloting the questions and presentation of physical literacy content. This helped to refine our approach and therefore supported the interview’s appropriateness and relevance and ensured that it was useful for capturing data from participants to answer the research questions. Prior to the initial interview, consenting participants were emailed the PL-C Quest user guide, the PL-C Quest assessment and additional information about physical literacy. During the initial interview, participants were introduced to the concept of physical literacy by explaining the APLF (Australian Sports Commission, 2020) and the PL-C Quest. Researcher AE then facilitated a semi-structured interview with individual participants that investigated what they knew about physical literacy and whether components of the APLF linked (or not) with occupational therapy concepts. Subsequent interviews were conducted with participants who also consented to trialling the PL-C Quest with at least one current client on their clinical caseload. Participants were asked to select a child to trial the PL-C Quest with and to observe and reflect on its administration, use and purpose within their practice, and overall relevance (or not) to occupational therapy. Both initial and subsequent interviews ranged from 15 to 60 min were digitally audio-recorded and transcribed verbatim.

### Data analysis

Reflexive thematic analysis was used to analyse the interview data. The six steps of Braun and Clarke’s (2022) thematic analysis method were applied by AE and discussed iteratively with all authors to ensure study rigour. This began with familiarisation with the data through transcribing the interview data and reading transcripts several times. Data were then coded inductively, and initial themes were generated from the initial codes and discussed amongst all authors. Following multiple team reviews of the themes and coded data, significant themes were identified, named and agreed upon as reflective of the data. Trustworthiness of the research was sought via regular debriefing and reflexive practice (Smith and McGannon, 2018). Investigator debriefing occurred across all stages of the research process and was amplified during data analysis between AE who coded the data and generated initial themes and the wider research team. Authors met regularly to discuss and challenge their interpretation of the data and to reflect on the influence of their own perspectives and world views, and how these might contrast with perspectives of the study participants and their clinical patients. The author team acknowledged the influence of their own critical realist perspective, which asserts that knowledge of reality is shaped by our perceptions and beliefs (Danermark et al., 2019). All authors had a health background, which spanned paediatric occupational therapy practice, motor skill development, physical literacy, physical activity, and qualitative research methods.

## Results

### Participant characteristics

Participants varied in their industry experience, from 3 to 26 years, and most were based in private practice. They worked with a range of neurotypical and atypical children, and their work included children with diagnoses of neurological, intellectual, psychosocial, physical/motor or development problems. Six of the eight consented to trial the PL-C Quest in practice which resulted in 12 children from ages 5 to 15 years completing the PL-C Quest with their occupational therapist. Both versions of the PL-C Quest were trialled in various settings, such as telehealth, in-clinic, at school and in-home sessions. Occupational therapists used the older child version of the PL-C Quest with four children (mean age of children completing the older child version was 12.00 years). One occupational therapist used the older child version of the PL-C Quest with a child aged 15 years which is outside the age range of the intended users for this tool. The occupational therapist considered their clinical reasoning and determined that this tool would be appropriate for this particular child to complete based upon the child’s disability and developmental age. There were eight children who completed the younger child version of the PL-C Quest (mean age of children completing the younger child version was 6.38 years). Participant characteristics are described in Table 1. The two participants who did not trial the PL-C Wuest in their clinical practice cited numerous reasons for not trialling; including (i) believing children on their caseload were unsuitable for use based on the child’s diagnosis, (ii) current workload capacity and not having the time and (iii) scope of practice limiting their ability to trial.

**Table 1.** Participant and Physical Literacy in Children Questionnaire (PL-C) trial characteristics.

Participant	Years of experience	Area of practice	Location	Age range of clients (years)	Number of interviews	Number of instrument trials completed	Age of child who completed trial
1	10+	Private	Regional	3–18	2	4	6 7 7 12^a^
2	10+	Private	Regional	0–8	2	2	5 7
3	0–5	Private	Regional	3–18	3	1	6
4	10+	Private	Regional	3–18	2	2	6 15^a^
5	0–5	Schools	Regional	3–18	2	0	–
6	10+	Private	Regional	3–18	2	1	12^a^
7	10+	Private	Urban	0–18	2	2	7 9^a^
8	5–10	Private	Regional	3–18	1	0	–

a Denotes Older Child Version PL-C Quest used.

### Themes

Four themes were developed from the data: Foreign tongue, Sounds very OT oriented, We need the child’s voice and Contemporary, useful, but not for all. The first two themes relate to the concept of physical literacy and occupational therapy practice, and the third and fourth themes relate to the use of the PL-C Quest in occupational therapy practice.

#### Foreign tongue

The first theme focused on understanding of the physical literacy construct. Most participants had not heard of physical literacy before participating in the study and were interested to learn more. Some occupational therapists understood the term *physical literacy* as ‘a person’s knowledge and understanding around physical health’ (Participant 1) and ‘how children would consider their own ability to participate in physical activity’ (Participant 7). As one occupational therapist said, ‘I know that kids need a lot of physical activity and opportunities to develop that, and then I wondered whether that [physical literacy] might fill a gap’. (Participant 2).

Discussion of the physical literacy framework revealed most physical literacy elements were assessed by occupational therapists in practice, but different terms were used, for example, *Participant 6* expressed: ‘we would probably use maybe different language’. Occupational therapists said some of the physical elements would be assessed informally on how they may impact children functionally. As one occupational therapist said: ‘I probably do look at all of those things, but through the lens of a functional task’ (Participant 4). Physical elements least typically considered in their assessments were ‘Cardiovascular Endurance’, ‘Muscular Endurance’, ‘Flexibility’ and ‘Reaction Time’. Participants expressed they would assess all the psychological elements, with some considering ‘Connection to Place’ as an environmental factor – rather than a psychological element. Participants also expressed they would assess all social elements, noting that they would not evaluate ‘Ethics’ specifically. Some reported they might consider observations and interactions to ‘look at things with the ethical perspective’ (Participant 4) and as ‘expected and unexpected behaviours’ (Participant 8). Most would assess the cognitive elements, but some said they would not consider ‘Tactics’. They noted they would use structured and unstructured interviews to gather subjective information on most elements from children and their parents.

After trialling the PL-C Quest in practice, participants were able to understand physical literacy terminology more clearly in its relevance to occupational therapy. One occupational therapist said:

I might not have necessarily categorised some of those questions into those particular areas. So, it was good for organising things like the psychological versus the cognitive. Some of those I’d probably all just lump in together and it would either be what I observed versus what’s reported and not necessarily break that down in the same way, so I thought that was interesting. But overall, I still see physical literacy as a relevant aspect to occupational therapy (Participant 1).

#### Sounds very OT oriented

The second theme illustrates a relationship between physical literacy and occupational therapy and highlights a familiarity between physical literacy and occupational therapy concepts. For example, one participant described physical literacy as ‘sounds very OT, isn’t it’ (Participant 4). Participants noted that physical literacy domains could relate to the Person, Environment and Occupational Performance Model (PEOP) (Baum et al., 2015), Model of Human Occupation (Taylor, 2017) and the Canadian Occupational Performance Measure (Law and Canadian Association of Occupational Therapists, 2005). Although few of the participants formally used occupational therapy models in their practice, and mostly used core concepts in their background thinking, including occupation as a means and an outcome, person/client/family-centred, client-directed, goal identification and setting and clinical reasoning. One occupational therapist described their approach as, ‘putting the child at the centre of their therapy and letting them be the experts on what they can and can’t do and what’s important to them’ (Participant 7).

Participants felt that the physical literacy domains were very occupational therapy oriented, but acknowledged it was an area of their practice that is not well covered and where there are currently limited resources. One occupational therapist said:

there are lots of potential links. It covers lots of areas that OT’s look at. The physical, psychological, social, and cognitive. And it’s really looking at how in functional terms, in a physical sense, it impacts on kids. So very OT oriented (Participant 6).

#### We need the child’s voice

The third theme involved the value of the child’s voice. All participants expressed the importance of subjective information gathering and self-reporting tools to support their practice. As one occupational therapist explained: ‘[self-reporting tools are] important for getting a holistic and collaborative understanding of the young person that you’re working with, to make sure that that person-centred care is happening’ (Participant 6). Participants noted that self-reporting tools are useful for gaining an understanding of a child’s insight into their own abilities and perception and being able to adapt interventions accordingly.

Participants discussed many benefits of using the PL-C Quest but an overarching highlight was hearing the child’s voice. As one occupational therapist said: ‘I think anything that gives the student or the child a voice definitely can benefit’ (Participant 2). When using the PL-C Quest in practice with a client, one participant expressed:

I think it actually was really nice for her to explain some of those barriers, in her words, and actually differentiate some of those things. And it was very much that perception of being lazy is what is talked about from the parent’s point of view and that not being her perception and that being really challenging to navigate (Participant 6).

Several participants highlighted a gap in current resources for self-reporting in children, expressing: ‘missing a child’s outlook on the activities if it’s important to them. But also, social, and cognitive components, they [existing assessments] purely just measure motor skills as they are, as we see them’ (Participant 1). Most participants indicated that there is not another comparable assessment presently used in this area of practice to gather information about a child’s physical capabilities that captures the child’s voice, expressing: ‘a lot of the assessments are either parent or therapist interpretations. And I mean, this is what it’s all for, this is the child’s voice. So that’s probably the main difference for me’ (Participant 2).

#### Contemporary, useful, but not for all

The fourth theme reflects participant perspectives on the feasibility of the PL-C Quest in their occupational therapy practice. After trialling the tool, participants described how it could be used to support and strengthen their practice as well as key considerations for determining when to use it. These are captured in two sub-themes: ‘A useful addition to the OT toolbox’ and ‘Considerations for practice’.

##### A useful addition to the OT toolbox

Before trialling the tool, participants reported that they could see the PL-C Quest used in practice, identifying a range of reasons including focusing on the child’s goals, expressing self-perceptions, gathering specific information, directing therapy and supporting clinical reasoning. Client-centred practice was an important reason for most occupational therapists when expressing their views on the PL-C Quest’s use in practice, with one occupational therapist highlighting that:

With client-centred practice, you want the goals to be able to be identified from the child as much as possible and using a subjective tool, such as [PL-C Quest] would be a good way for children to say, ‘yes I don’t participate but I want to’ and could be a really meaningful and nice way for them to provide goals themselves (Participant 3).

After trialling the PL-C Quest, participants still felt that it was a relevant tool in occupational therapy practice. The time to complete the PL-C Quest varied from 10 to 20 min, with an average of 17 min. Participants appreciated the presentation and administration of the PL-C Quest; noting the picture-based questions and simple format made the assessment easy to follow and simple to administer. Compared to other assessments, participants said the PL-C Quest is ‘a lot more with it in this day and age’ (Participant 3) with images that capture attention and are relevant to the Australian context, with one participant stating ‘a lot of [assessments] are either American based or sort of European based. And it’s sometimes not very relevant with the wording’ (Participant 3).

Participants felt that the PL-C Quest used a plain language approach, and they appreciated the visual-based questions as it allowed them to build rapport and gather information with children experiencing mental illness or developmental challenges where they otherwise would have had difficulty building trust. Although, most participants felt that some children did not answer the questions accurately, often choosing the positive image on the left-hand side or an image that reflected what they enjoyed doing rather than their capabilities. One participant expressed that it would be helpful to adapt the questions into a card format so that the image order could be changed, stating:

And the other thing I probably would have done is mixed them. . . My [client] was just picking what they worked out what the box was that they thought was the ‘good’ box. So I would have put the negative one on the other side. . . just to make sure that they were thinking about the question (Participant 2).

Participants thought the PL-C Quest could be suitable in settings of paediatric occupational therapy, allied health, schools, early childhood and community-based groups. One participant described: ‘it’s not something that I would have thought of as useful in my setting, but it definitely is probably something that I would think of as useful now’ (Participant 6). Participants felt that the PL-C Quest would be suitable for primary school-age children without a diagnosis who are experiencing social/emotional challenges, unclear participation barriers, report challenges participating in physical activity or have limited leisure outlets to guide goal setting. Participants used information gathered from the PL-C Quest in several ways including to educate parents, develop goals, understand the child’s perceptions, and to guide intervention. One participant explained how she talked to a parent about why using the PL-C Quest was a helpful tool in her practice, reflecting:

I just kind of explained it as that ‘it kind of gives us some more information about what so-and-so thinks about, you know, participating, and the skills needed to be active and partake in different activities. It gives us, me more insight into their insights’ (Participant 7).

Another participant discussed how using the PLC-Quest could assist in identifying occupational performance issues and goals:

It’s helped me understand that the family are at least interested in pursuing more information in physical literacy, which does help me sort of almost steer goals. So it’s actually quite useful because it does, it opens up another, another avenue to explore, where previously, it’s, you know, it’s somewhat generic, you know, self-care, fine motor etc. (Participant 3).

Most participants stated that the PL-C Quest benefited their practice, and they would recommend the PL-C Quest to other paediatric occupational therapists to use in practice. Several participants were not able to see the PL-C Quest as useful in their scope of practice but found it useful upon trial in practice. Participants reported: ‘It helped me gather more information about the child than I would have’ (Participant 4) and ‘It was actually a really nice way to open up conversation with the young person about what was challenging’ (Participant 6).

##### Considerations for practice

Some participants questioned whether the PL-C Quest would be suitable for children with severe physical disabilities, limited insight or self-concept, significant behavioural challenges, moderate intellectual disability, and autistic children requiring very substantial support. The children that trialled the PL-C Quest in practice responded well and found the pictures engaging; however, some children had difficulty understanding the meaning of some images and required further explanation. For example, some children with language delays and poor social and pragmatic language, appeared to misinterpret some questions and took a more black and white literal approach. The occupational therapists using the PL-C Quest with autistic children suggested typical clinical symptoms such as literal thinking and decreased insight into self-perception and self-awareness made visual-based questions and language challenging to interpret. These therapists reported varying results, noting challenges experienced were due to literal thinking and reduced ability to read social situations from images.

One participant described her experience with a child with a diagnosis of autism spectrum disorder who took Question 10 quite literally:

The item says, some children are pretty good when strong muscles are needed such as picking up a rock. They were very literal about, it had to be a rock that they had to pick up: ‘Like, when I go to my friend’s house, I can pick up the rocks in their backyard.’ And I was like, what about other times when you need to use strong muscles? And then they’re like: ‘Oh, no, not really’ (Participant 7).

Another participant commented on their experience using the PLC-Quest with children diagnosed with autism, noting that there were instances where the PLC-Quest worked well:

Some of the autistic children did quite well, and perhaps they were the children that had more experience [with occupational therapy], so have had more intervention. And that were more comfortable, they had better interoception and reflective abilities and some self-regulation (Participant 1).

Participants noted that changes could be made to some questions and images to accommodate children with a disability, including high contrast for visually impaired, adjustments to images and language to increase understanding for children with difficulty interpreting social situations and literal thinking. One participant expressed:

I can see how a disability version could be created, that’s more therapy based. So, I think the structure, the way it’s done and completed, all the groundwork is there. . . I think it can be adapted for children with additional needs and more for therapists, I can see its potential there (Participant C).

Whilst all participants would recommend the PL-C Quest to other paediatric occupational therapists to use in practice, some emphasised the need for consideration of clinical reasoning for each child. Most agreed that further training and an information session would be appropriate on how and when to use the PL-C Quest and information gathered. Overall, all participants agreed that there is a place for the PL-C Quest in paediatric occupational therapy.

## Discussion

This is the first study to explore paediatric occupational therapists’ perspectives on physical literacy and its assessment in children. We sought to investigate occupational therapists’ knowledge of physical literacy and whether occupational therapists perceived a relationship between physical literacy and occupational therapy. This study also investigated the feasibility of the PL-C Quest with paediatric occupational therapists and whether this assessment tool is a valid self-assessment for paediatric clients.

Exploring the perspectives of paediatric occupational therapists in relation to physical literacy in healthcare practice is valuable as occupational therapists are skilled in considering a holistic view of their clients and various factors may influence a client’s participation (Cordier et al., 2016). Most occupational therapists participating in this study had a low understanding of *physical literacy*, noting they were unfamiliar with terminology and used different language to describe physical literacy concepts. Occupational therapists wanted to learn more to support their clients, recognising a gap in their understanding and consistent with the lack of literature relating to physical literacy in occupational therapy practice. This suggests that occupational therapists value further education and recognise continuing professional development as not only a requirement of maintaining their registration but also enabling them to address gaps in their practice to improve client outcomes and enhance clinical reasoning (Shafaroodi et al., 2017).

After trialling the PL-C Quest and breaking down the language used, occupational therapists could understand physical literacy terminology more clearly in its relevance to occupational therapy. Occupational therapists actually assessed most elements of the physical literacy domains in their practice; however, they tended to use different terminology in describing the various physical literacy concepts. For example, as part of their assessment process, occupational therapist participants tended to focus more on how and why a child participates in activity rather than the skills needed to perform that activity. In general, occupational therapists use both top-down perspectives with a focus on participation and subjective view of occupational engagement, as well as bottom-up approaches where the focus might be more directed at assessing or addressing deficits in skills and capabilities (Vas et al., 2021). This suggests that occupational therapists valued the PL-C Quest as it presents as and can be utilised as a top-down, occupation-focused participation assessment.

Occupational therapists acknowledged the relevance of the physical literacy domains (physical, psychological, social and cognitive) in occupational therapy models. Although most occupational therapists did not formally state the use of structured models in their practice, they described core concepts such as person-centred, client-centred and client-directed which stem from various occupational therapy theory (Baum et al., 2015; Taylor, 2017) indicating unconscious use. This demonstrates the overlap between occupational therapy and physical literacy concepts and speaks to the value of incorporating physical literacy within occupational therapy practice. Occupational therapists felt the self-assessment nature of the PL-C Quest supports these core concepts in gaining a holistic view of the child to guide intervention; this finding was consistent with Barnett et al. (2022a, 2022b) in the context of public health. Similar to the PEOP model (Baum et al., 2015), the APLF (Australian Sports Commission, 2020) breaks down the person and environment factors (e.g. physical/motor, psychological, cognitive and social) to gain an understanding of the strengths and barriers that may impact participation in occupations involving physical activity. Using the PL-C Quest, occupational therapists were able to look at the person factors more comprehensively to understand barriers of motivational participation for engaging in physical activity.

As paediatric occupational therapists work with children to increase or maintain participation in occupations, gaining an understanding of barriers such as components of physical literacy can support practice. Previous literature notes physical literacy as a determinant of participation in physical activity (Cairney et al., 2019) and occupational therapists highlighted that they often work with children reluctant to participate in physical activity due to unclear barriers. This is consistent with literature (Shields and Synnot, 2016) which suggests children with a disability engage in less physical activity than typically developing children. Occupational therapists noted that children should be participating in physical activity more regularly, consistent with physical activity guidelines (World Health Organization, 2020), further suggesting that the two lines of work share common ground.

Occupational therapists value subjective information gathering and self-reporting tools to support their practice; this is consistent with literature that suggests understanding a child’s subjective experience is essential to providing client-centred therapy (Cordier et al., 2016). Self-assessment measures promote opportunities for children to become an integral part of their own therapy, allowing them to voice their concerns and collaborate with an occupational therapist to establish goals (Cordier et al., 2016; Greco et al., 2016). Occupational therapists acknowledged ‘the child’s voice’ was the main benefit of this tool. Occupational therapists noted the child often has a different world view than their parents and gaining insight into the child’s perspective was often missed in formal assessments of physical capabilities in practice. There is uncertainty over how a child can determine their needs and goals relating to occupational performance with many ‘self-report’ tools actually parent- or teacher-reported (Greco et al., 2016), therefore, gathering subjective information from children should be a focus of paediatric occupational therapists. Occupational therapists who used the PL-C Quest highlighted a need for more formal tools to address this need.

Occupational therapists must choose self-report measures with established validity, reliability and clinical utility to guide holistic interventions for children (Cordier et al., 2016). Whilst the PL-C Quest has good evidence of psychometrics (Barnett et al., 2022a, 2022b), it has only been tested with typically developing children. In contrast, occupational therapists working in paediatrics will most commonly work with children with various health conditions and clinical diagnoses impacting their development. Therefore, an individual occupational therapists clinical reasoning based upon numerous factors (including the child’s presentation, the clinician’s experience and relevant evidence and theory) was found to be an important consideration for utility of the PL-C Quest in practice. To support this, most participants agreed that training and further information would be appropriate on how and when to use the PL-C Quest and the subsequent information gathered; suggesting occupational therapists may need guidance to unpack *physical literacy* and how to use information gathered from PL-C Quest.

### Implications for practice

The findings from this study provide preliminary evidence to support a deeper understanding of the relationship between physical literacy constructs and occupational therapy practice. Results confirm the importance of understanding the child’s perspective and hearing the child’s voice, which is pertinent to both child- and family-centred practice in paediatric occupational therapy. Self-report is common in occupational therapy practice and the findings speak to the applicability and importance of using tools to gather subjective information important for understanding a child’s participation more accurately. Furthermore, with a gap in how physical literacy is perceived and utilised in healthcare practice, the findings support the utility of physical literacy and the PL-C Quest in professions and settings beyond education and health promotion. Finally, this research contributes to the wider physical literacy evidence base and supports ongoing use of the PL-C Quest in physical literacy.

### Limitations

The research team’s combined experience of paediatric occupational therapy, qualitative research and development of PL-C Quest enhanced the research design and thematic analysis of data. Due to limited recruitment and trial timeline, time allowing for participants to use the assessment in practice was reduced. As this was a small qualitative study, participants were recruited from a specific geographical area for convenience, indicating the results may not be generalisable to other populations. Finally, this study did not interview children or observe real-time assessment with occupational therapists; therefore, it is unknown how much of the perspectives reflect actual practice. However, occupational therapists who participated in the study were experienced in paediatric practice and have experience to provide strong clinical reasoning and reflection to provide in-depth data.

### Recommendations for future research

Future research is required to add to the limited evidence regarding occupational therapy and physical literacy. Further investigation of the feasibility and utility of the PL-C Quest in clinical populations receiving occupational therapy intervention would be useful to determine appropriate use for specific health conditions. This could lay a foundation for subsequent research investigating potential adaptations to the PL-C Quest based on clinical diagnosis and relevance specifically for occupational therapy.

## Conclusion

This study aimed to investigate occupational therapists’ perspectives of physical literacy and the feasibility of the PL-C Quest in occupational therapy practice. Physical literacy was not a familiar construct amongst participants, but upon in-depth discussion appeared to be relevant to occupational therapy practice and aligned with several occupational therapy methods. Six paediatric occupational therapists trialled the PL-C Quest in clinical practice with children aged between 5 and 15 years. Findings indicated the PL-C Quest has the potential to fill an important gap in occupational therapy clinical practice by capturing the child’s own perspective. Feasibility of the PL-C Quest in paediatric occupational therapy practice was supported, provided clinical reasoning considerations for each client are examined. There is a need for further research to understand physical literacy and assessment for children with a clinical diagnosis.

**Key findings**

- The concept of physical literacy is relevant to paediatric occupational therapy practice.
- Self-reporting tools are valuable for promoting the child’s voice in assessment.
- The PL-C Quest is feasible for use in paediatric occupational therapy to understand barriers to participation in physical activity.

**What the study has added**

This study has contributed to occupational therapy practice by highlighting the importance of capturing the child’s voice through subjective assessment. Furthermore, it demonstrates occupational therapists engage with physical literacy concepts and use these readily to inform their clinical practice with paediatric clients, supporting interdisciplinary collaboration.
